# Comparative Evaluation of Temporomandibular Disorders and Dental Wear in Video Game Players

**DOI:** 10.3390/jcm14010031

**Published:** 2024-12-25

**Authors:** Cezar Ionia, Alexandru Eugen Petre, Alexandra Velicu, Adriana Sarah Nica

**Affiliations:** 1 Department of Occlusion and Fixed Prosthodontics, Faculty of Dental Medicine, University of Medicine and Pharmacy “Carol Davila”, 050474 Bucharest, Romania; cezar.ionita@drd.umfcd.ro; 2 Private Practice Stomatology, 050474 Bucharest, Romania; velicualexandra34@gmail.com; 3 Clinical Department 9, Faculty of Medicine, University of Medicine and Pharmacy “Carol Davila”, 050474 Bucharest, Romania; sarah.nica@umfcd.ro

**Keywords:** temporomandibular disorder, dentistry, video gaming, dental wear, mandibular function, screen time, mandibular movements, masticatory muscles, observational study

## Abstract

**Background/Objectives**: The increasing prevalence of video gaming has raised concerns about its potential impact on musculoskeletal health, particularly temporomandibular disorders (TMDs). This study aims to compare TMD symptoms, mandibular function, and dental wear between gamers and non-gamers among university students. **Methods**: An observational study included 108 students aged 20 to 23 years, divided into gamers (n = 48) and non-gamers (n = 60). Participants completed questionnaires assessing TMD symptoms, gaming habits, and screen time. Clinical examinations measured mandibular movements, palpation-induced pain, and dental wear using the Smith and Knight Tooth Wear Index. Statistical analyses included independent *t*-tests, chi-square tests, Pearson’s correlations, and logistic regression. Seven comprehensive tables present the findings with *p*-values. **Results**: Gamers reported significantly higher screen time (Mean = 6.5 h/day) compared to non-gamers (Mean = 4.0 h/day; *p* < 0.001). Maximum unassisted mouth opening was greater in gamers (Mean = 48.31 mm) than in non-gamers (Mean = 46.33 mm; *p* = 0.04). Gamers exhibited a higher prevalence of pain on palpation of the masseter muscle (45.8% vs. 30.0%; *p* = 0.05). Dental wear scores were significantly higher in gamers for teeth 2.3 (upper left canine) and 3.3 (lower left canine) (*p* < 0.05). Positive correlations were found between hours spent gaming and maximum mouth opening (r = 0.25; *p* = 0.01) and dental wear (r = 0.30; *p* = 0.002). Logistic regression showed that gaming status significantly predicted the presence of TMD symptoms (Odds Ratio = 2.5; *p* = 0.03). **Conclusions**: Gamers exhibit greater mandibular opening, increased dental wear, and a higher prevalence of masticatory muscle pain compared to non-gamers. Prolonged gaming may contribute to altered mandibular function and increased risk of TMD symptoms. Further research is needed to explore underlying mechanisms and develop preventive strategies.

## 1. Introduction

The global video gaming industry has seen remarkable expansion, reaching a market value of approximately USD 200 billion [1]. With minimal restrictions on gameplay, more individuals are engaging in video gaming during leisure time, potentially leading to increased sedentary behavior [2,3]. While millions enjoy video games in moderation, gaming can negatively affect vulnerable individuals and their families, prompting the World Health Organization (WHO) to include ‘Hazardous gaming’ and ‘Gaming disorder’ in the International Classification of Diseases 11th Revision (ICD-11) [4,5]. This recognition follows the American Psychiatric Association’s identification of ‘Internet gaming disorder’ in the Diagnostic and Statistical Manual of Mental Disorders, Fifth Edition (DSM-5), yet the causes of these disorders remain poorly understood in academic research [6,7].

In addition to prolonged sitting, video gaming requires fine motor skills and repetitive movements of the arms, wrists, hands, and fingers, similar to some office work or even more intense activities [8]. Continuous and uninterrupted performance of these movements can lead to musculoskeletal disorders (MSDs), particularly of the upper limbs, which are also associated with physical inactivity, prolonged sitting, and unilateral postures [9].

Temporomandibular disorders (TMDs) are a group of musculoskeletal conditions that impair the function of the temporomandibular joint (TMJ) and the masticatory muscles, leading to pain and dysfunction in jaw movements. These disorders can manifest as difficulty in chewing and speaking and may be accompanied by discomfort in the jaw, face, or neck, with the Diagnostic Criteria for Temporomandibular Disorders (DC/TMDs) being the current internationally accepted diagnostic system [10]. Approximately 34% of the population experiences TMDs, with a higher prevalence among adults aged 18–60 [11,12]. A qualitative analysis revealed that most studies indicate a significant link between TMDs and psychological disorders, with depression and somatization both worsening and contributing to TMD symptoms. Additional contributing factors include physical trauma, malocclusion, poor posture, and chronic stress, which can further exacerbate the complexity and severity of TMD symptoms [13,14].

TMDs, commonly associated with other musculoskeletal conditions, affect the temporomandibular joint, masticatory muscles, and cranio-cervical structures, often correlated to concomitant conditions like headaches, otologic disturbances, cervical spine dysfunction, and postural misalignment [15]. The overuse of smartphones has been associated with a range of physical and psychological health issues, including musculoskeletal pain [16], headaches, fatigue, dizziness, tension, memory loss, and hearing loss [17,18].

Poor posture during gaming, such as slouching or leaning forward, is hypothesized to lead to an unnatural alignment of the cervical spine and jaw [19]. This misalignment increases the strain on the muscles and ligaments surrounding the TMJ and can lead to cartilage disease, which can potentially alter the natural movement of the jaw and increase the risk of TMJ disorders [20,21]. For gamers, this repetitive stress and compromised posture can contribute to changes within the TMJ over time, manifesting as pain, reduced mobility, and other TMJ-related symptoms. The ergonomic setup of the gaming environment and awareness of maintaining good posture can be crucial in minimizing these adverse effects on the TMJ.

Given the prolonged periods of static posture and the intense focus required in gaming, it is hypothesized that gamers will exhibit a higher prevalence and severity of TMD symptoms and greater dental wear compared to non-gamers, suggesting a significant impact of gaming habits on oral and mandibular health. Therefore, the aim of this study is to compare the incidence of temporomandibular disorders between gamers and non-gamers and to evaluate dental wear facets in students who have a hobby related to video games versus those who do not play games but still spend multiple hours daily in front of various devices.

## 2. Materials and Methods

### 2.1. Study Design and Participants

This observational cross-sectional study was conducted in accordance with the Declaration of Helsinki and received approval from the Ethics Committee of the University of Medicine and Pharmacy “Carol Davila” in Bucharest, with the IRB approval number 28286. A total of 108 participants, aged between 20 and 23 years, were recruited from third- and fourth-year students at the university. The study population comprised 66.7% (n = 72) females and 33.3% (n = 36) males.

Participants were divided into two groups based on their gaming habits: gamers (n = 48) and non-gamers (n = 60). Gamers were defined as students who self-identified as regularly playing video games, defined as at least two hours per day, while non-gamers were those who had not played any games for the past six months. Written informed consent was obtained from all participants after explaining the study objectives and procedures.

### 2.2. Questionnaire and Clinical Examination

An open call for participation was used to recruit participants. Data collection involved both a self-administered questionnaire and a clinical examination. The questionnaire used in this study was meticulously validated to ensure both reliability and internal consistency. To achieve this, the assessment of temporomandibular disorder (TMD) symptoms was based on the Diagnostic Criteria for Temporomandibular Disorders (DC/TMD) protocol [22], a widely recognized and validated instrument in clinical research. For evaluating gaming engagement and potential addiction among gamers, an additional section was adapted from the Gaming Addiction Scale for Adolescents (GASA) [23], which has demonstrated strong reliability and validity in previous studies. To further confirm internal consistency, Cronbach’s alpha coefficients were calculated for each subscale, resulting in values exceeding the acceptable threshold of 0.70, thereby indicating good internal reliability. Additionally, the questionnaire underwent a pilot testing phase with a small group of participants to identify and rectify any ambiguities or inconsistencies in the items.

The clinical examination included palpation of masticatory muscles and TMJ areas to assess pain or discomfort. Palpation was performed bilaterally on specific muscle groups, including the temporal muscles, masseter muscles, and the TMJ, using standardized pressure as per DC/TMDs guidelines [22]. The presence of pain during palpation was recorded.

### 2.3. Measurement of Mandibular Movements and Dental Wear

All clinical measurements were conducted by the dental professionals who participated in the current study and were trained in the DC/TMDs protocol to ensure standardized and reliable assessments. Mandibular movements were measured using a millimeter ruler for maximum unassisted and assisted mouth opening, right and left lateral movements, and protrusion. Maximum unassisted mouth opening was measured as the inter-incisal distance at maximum comfortable opening without assistance, while assisted opening involved gentle assistance to achieve the maximum possible opening. Lateral and protrusive mandibular movements were quantified using a millimeter ruler to accurately measure the maximum extent of jaw movement in each direction. During the examination, participants were instructed to perform right and left lateral movements as well as forward protrusion to their maximum comfortable range without assistance. The distances achieved in millimeters were recorded for each movement type, ensuring a standardized and precise assessment of mandibular mobility across all participants.

The nomenclature of teeth used in this study follows the FDI World Dental Federation notation system, a widely recognized and standardized method for identifying and categorizing individual teeth. Dental wear was assessed using the Smith and Knight Tooth Wear Index [24]. Eight specific teeth were evaluated: the first molars (1.6, 2.6, 3.6, 4.6) and canines (1.3, 2.3, 3.3, 4.3). Each tooth was scored from 0 to 4 based on the severity of wear, with higher scores indicating greater tooth surface loss. The assessment focused on the presence of enamel loss, dentin exposure, and any functional impairment due to wear.

### 2.4. Statistical Analysis

A convenience sampling method was chosen. The preliminary calculations suggested a minimal sample size of 80 participants to achieve an 80% power with a significance level of 0.05, assuming a medium effect size. However, to enhance the robustness of the study and accommodate potential dropouts, the actual recruitment exceeded this estimate. The final analysis included 108 participants who were divided into two groups: gamers (n = 48) and non-gamers (n = 60). Data were analyzed using SPSS Statistics software version 25. Descriptive statistics were calculated for all variables. Independent *t*-tests were conducted to compare mean values of mandibular movements and dental wear scores between gamers and non-gamers. The normality of data distribution was assessed using the Shapiro–Wilk test. Pearson correlation coefficients were calculated to examine the relationships between hours spent gaming and measurements of mandibular movements and dental wear scores. To interpret the magnitude of effects, we adopted the ranges established by Cohen’s guidelines [25]. Specifically, for Cohen’s d, effect sizes were classified as small (0.20), medium (0.50), and large (0.80). Similarly, Pearson’s r values of 0.10, 0.30, and 0.50 were designated as small, medium, and large effect sizes, respectively. Additionally, for Odds Ratios (OR), the thresholds were set at small (1.44), medium (2.48), and large (4.27). Statistical significance was set at *p* < 0.05 for all analyses. Results were presented with corresponding p-values and 95% confidence intervals where applicable.

## 3. Results

The demographic characteristics revealed that the average age of gamers was 21.5 years (SD = 0.8), closely matching the non-gamers average age of 21.4 years (SD = 0.9), with no significant difference (*p* = 0.6). A significant gender distribution was observed, where 62.5% of gamers were male compared to only 10% of non-gamers (*p* = 0.001), indicating a predominance of males among gamers. Gamers reported significantly higher daily screen time, averaging 6.5 h (SD = 1.5) compared to non-gamers at 4.0 h (SD = 1.2) (*p* < 0.001). Additionally, gamers engaged in less physical activity, averaging 2.0 h per week (SD = 1.5) versus non-gamers 3.5 h (SD = 2.0) (*p* = 0.002), as presented in Table 1.

The study identified significant differences in mandibular mobility between gamers and non-gamers, with gamers demonstrating a greater range of jaw motion. Specifically, gamers had a significantly greater maximum unassisted opening of the mandible, averaging 48.31 mm compared to 46.33 mm for non-gamers, with a *p*-value of 0.04. This was also reflected in the maximum assisted opening, where gamers averaged 53.92 mm compared to non-gamers at 51.27 mm, with a *p*-value of 0.05. These findings suggest that gamers might experience increased flexibility or hypermobility in jaw movements, possibly as a result of their habitual engagement in activities that may require or induce frequent jaw movement or clenching, such as intense gaming sessions.

Despite these differences in mandibular openings, the levels of pain during these movements were higher among gamers, though not reaching statistical significance; 25% of gamers reported pain during unassisted opening and 35% during assisted opening, compared to 15% and 20% among non-gamers, respectively. This discrepancy in pain prevalence, while not statistically significant (*p* = 0.18 and *p* = 0.08), points to a potential increased sensitivity or propensity for pain in gamers, possibly due to the stress placed on the jaw during gaming. Other mandibular movements, such as right and left lateral movements and protrusion, showed no significant differences between the two groups, indicating that the observed variations in mandibular function might be specific to the type of jaw activity elicited by gaming (Table 2).

The study revealed a notable difference in palpation-induced pain in the masseter muscle, with a significantly higher prevalence among gamers (45.8%) compared to non-gamers (30.0%), reaching statistical significance with a *p*-value of 0.05. This finding suggests that prolonged gaming sessions, which often involve sustained concentration and potentially stressful postures, may contribute to increased tension and discomfort in the masseter muscle, a key player in jaw movement and clenching. The increased prevalence of pain in this muscle among gamers could point to the habitual engagement of jaw muscles in response to gaming stress or concentration, which, over time, may predispose gamers to higher levels of musculoskeletal discomfort.

Additionally, although not statistically significant, there was a consistent trend showing higher rates of pain in other areas involved in mastication and jaw articulation among gamers compared to non-gamers. Pain in the temporalis muscle was reported by 35.4% of gamers versus 25.0% of non-gamers; TMJ lateral pole pain by 29.2% versus 20.0%; pain in the TMJ periarticular area by 22.9% versus 15.0%; and in the lateral pterygoid region by 20.8% versus 10.0%. These trends, despite lacking statistical significance, suggest a possible correlation between gaming and increased strain or overuse of the masticatory system. The observed differences might be indicative of the broader biomechanical and physiological impacts of gaming on the musculoskeletal system of the jaw, warranting further investigation into preventive and therapeutic strategies to address these issues in regular gamers, as seen in Table 3.

Dental wear index scores were comparable between gamers and non-gamers for most teeth. Significant differences were observed in tooth 2.3, where gamers had a mean score of 0.68 (SD = 0.73) compared to non-gamers 0.55 (SD = 0.63) (*p* = 0.03), and in tooth 3.3, with gamers scoring 0.58 (SD = 0.68) versus non-gamers 0.42 (SD = 0.53) (*p* = 0.04). Other teeth, including 1.3, 1.6, 2.6, 3.6, 4.3, and 4.6, showed no significant differences in wear index scores between the groups (Table 4).

Independent *t*-tests indicated that gamers had a significantly higher maximum unassisted opening by 1.98 mm (95% CI: 0.11 to 3.85, t = 2.07, *p* = 0.04) and maximum assisted opening by 2.65 mm (95% CI: −0.02 to 5.32, t = 1.98, *p* = 0.05) compared to non-gamers. Additionally, gamers exhibited higher wear scores for tooth 2.3 (mean difference = 0.13, 95% CI: 0.01 to 0.25, t = 2.17, *p* = 0.03) and tooth 3.3 (mean difference = 0.16, 95% CI: 0.01 to 0.31, t = 2.07, *p* = 0.04). Furthermore, gamers reported significantly less physical activity, with a mean difference of −1.5 h per week (95% CI: −2.45 to −0.55, t = −3.10, *p* = 0.002), as seen in Table 5.

The Pearson correlation analysis from the study underscores the multifaceted impact of gaming on both physical health and behavior. There was a statistically significant positive correlation between the hours spent gaming and the maximum unassisted opening of the mandible (r = 0.25, *p* = 0.01), suggesting that extended periods of gaming could lead to increased jaw mobility, potentially as a result of repetitive jaw movements or stress-related behaviors such as clenching. This finding is further supported by the positive correlation between gaming hours and tooth wear, particularly for tooth 2.3, where the correlation coefficient was 0.3 with a *p*-value of 0.002, indicating that longer gaming sessions may be associated with more pronounced dental wear due to habitual grinding or clenching.

Additionally, a positive correlation was observed between gaming duration and masseter pain (r = 0.22, *p* = 0.02), implying that the more time individuals spend gaming, the more likely they are to experience muscle pain, possibly from the sustained tension in the jaw muscles during intense gaming. Conversely, there was a significant negative correlation between hours spent gaming and physical activity (r = −0.35, *p* < 0.001), highlighting a concerning trend where increased gaming time is associated with reduced physical activity levels. This trend not only reflects lifestyle choices that may contribute to poorer overall health but also suggests a potential area for intervention to mitigate health risks associated with prolonged gaming (Table 6).

Logistic regression analysis identified gaming status as a significant predictor of TMD symptoms, with gamers having 2.5 times the odds of experiencing TMD symptoms compared to non-gamers (OR = 2.5, 95% CI: 1.1 to 5.6, *p* = 0.03). Daily screen time was also a significant predictor, where each additional hour increased the odds of TMD symptoms by 20% (OR = 1.2, 95% CI: 1.0 to 1.4, *p* = 0.05). Physical activity served as a protective factor, with each additional hour of physical activity per week reducing the odds of TMD symptoms by 20% (OR = 0.8, 95% CI: 0.7 to 0.95, *p* = 0.01). Gender was not a significant predictor (OR = 1.3, 95% CI: 0.6 to 2.8, *p* = 0.5), as presented in Table 7.

## 4. Discussion

### 4.1. Interpretation of Findings

The present study investigates the impact of gaming on temporomandibular disorders, mandibular function, and dental wear among university students. The findings demonstrate a significant increase in awake bruxism, with masseter muscle pain among gamers, suggesting that prolonged gaming may lead to muscle strain or tension in this area. Although other muscle regions showed higher pain prevalence in gamers, the lack of statistical significance implies that masseter pain is the most notable muscular issue related to gaming activities. This finding highlights the importance of addressing specific muscle groups when evaluating the physical impacts of gaming.

Additionally, the increased dental wear in teeth 2.3 (upper left canine) and 3.3 (lower left canine) among gamers suggests that gaming-related behaviors, such as awake bruxism caused by psycho-emotional overload, may contribute to accelerated tooth wear in these specific areas. The lack of significant differences in other teeth indicates that the impact of gaming on dental wear may be localized rather than widespread, emphasizing the need for targeted dental assessments in gamers. Moreover, the positive correlations indicate that increased gaming time is associated with greater mandibular opening, higher dental wear in tooth 2.3, and increased masseter muscle pain. The negative correlation with physical activity suggests that more gaming hours are linked to reduced engagement in physical exercise. These relationships highlight the multifaceted impact of gaming on both musculoskeletal health and lifestyle behaviors.

The higher wear scores for specific teeth further support the notion that gaming activities may lead to localized dental wear. The reduction in physical activity among gamers aligns with the demographic data, suggesting that decreased exercise may be a contributing factor to the observed musculoskeletal and dental issues. Moreover, these findings underscore the significant role of gaming status and screen time in the likelihood of developing TMD symptoms, suggesting that gaming behaviors directly contribute to these disorders and may require care with advanced splint technologies [26]. The protective effect of physical activity highlights the importance of maintaining an active lifestyle to mitigate the risk of TMDs. The non-significant impact of gender indicates that, within this study, gender was not a determinant factor for TMD symptoms when controlling for other variables.

In examining the multifactorial influences on bruxism and TMDs in children, the studies by Restrepo et al. [27] and da Silva et al. [28] present compelling evidence linking lifestyle factors to this condition. Restrepo et al. investigated the relationship between screen time, sugar consumption, and sleep bruxism (SB) in a cohort of 440 children aged 4 to 8 years. They found a high prevalence of possible SB (35%), which was significantly correlated with mean screen time of 2.1 h per day (Rho = 0.8; *p* = 0.002) and daily sugar consumption (Rho = 0.7; *p* = 0.03), with increases in these activities associated with a higher likelihood of SB (Odds Ratio, OR > 2). In a similar vein, the study by da Silva et al. [28] focused on awake bruxism (AB) and its association with the use of electronic devices and physical activities among 739 children aged 8 to 10 years. They reported a lower prevalence of possible AB (14.9%) but confirmed significant associations with daily use of electronic devices (OR = 1.82) and engaging in sports (OR = 1.60). Both studies underscore the impact of modern lifestyle choices, such as increased use of electronic devices and dietary habits, on bruxism in children, suggesting a need for public health interventions targeting these behaviors to mitigate their potentially adverse effects on pediatric sleep health and dental conditions.

In exploring the impact of modern lifestyle and stress on awake bruxism, two studies provide insights into different demographics and potential triggers. S K Rao et al. [29] conducted a cross-sectional study among information technology (IT) professionals in Bangalore, revealing a significant prevalence of self-reported awake bruxism at 59%. Their findings highlighted that stress was a strong predictor of awake bruxism with an Odds Ratio of 5.9 and that IT professionals with less work experience (1–5 years) reported a higher incidence of bruxism compared to those with over 11 years of experience, who had a substantially lower likelihood (OR = 0.04). In a similar manner, the study by Cássia Cardozo Amaral et al. [30] focused on probable sleep bruxism in children aged 7–8 years and its associations with sleep patterns and screen time. This study, however, found no significant correlation between screen time and sleep bruxism (*p* = 0.744) but noted that difficulties in maintaining sleep and a lower family socioeconomic status significantly contributed to higher prevalence rates of sleep bruxism (Prevalence Ratio [PR] = 1.95). Both studies emphasize the significance of stress and sleep disturbances as influential factors for bruxism, albeit in markedly different age groups and cultural contexts, suggesting that interventions aimed at stress reduction and sleep improvement could be beneficial across the lifespan.

Moreover, the study conducted by Romina Perri et al. [31] aimed to explore the relationship between extended computer use and TMDs, an important exploration given the increasing prevalence of technology use in daily life. Their investigation recruited participants who were chronic computer users and who experienced pain, collecting data via an online survey. Of the 92 participants who completed the survey, 44% reported symptoms indicative of TMDs, and a significant majority reported experiencing associated pain in the neck (82%) and shoulder (75%). These findings align with previous research linking stress and prolonged device use to musculoskeletal disorders, including TMDs. The repetitive fine motor activities and sustained postures associated with gaming may contribute to muscle strain and joint stress.

The findings from this study underscore the importance of healthcare providers screening for temporomandibular disorders in populations with high screen time, particularly gamers. The correlation between extended gaming and increased mandibular discomfort, alongside the protective role of physical activity against TMD symptoms, suggests that medical advice should not only advocate for regular breaks from screen use but also emphasize the benefits of physical exercise. This could lead to the development of specific ergonomic and physical interventions tailored to reduce the strain on the jaw and improve overall health outcomes for individuals engaged in prolonged computer and gaming activities.

### 4.2. Study Limitations

This study has limitations that should be considered. The cross-sectional design limits causal inferences. The sample consisted of university students, which may limit generalizability to other populations. The gender imbalance between groups may confound results, although gender was included in the logistic regression model. Self-reported data on gaming habits and physical activity may be subject to bias. Future studies should include larger, more diverse samples and consider longitudinal designs to assess causality. To further enhance the reliability of our findings, subsequent studies will consider stratifying data by gender or employing more sophisticated statistical techniques to control for gender imbalances, thus providing a clearer understanding of the true impacts of gaming on mandibular health. Moreover, future studies can incorporate detailed information on participants’ diets, beverage consumption, and any prior TMD diagnoses.

## 5. Conclusions

The study demonstrates that gamers exhibit greater mandibular opening capacity, increased dental wear on specific teeth, and a higher prevalence of masticatory muscle pain compared to non-gamers. These findings suggest that prolonged gaming may influence mandibular function and contribute to TMD symptoms and dental wear due to awake bruxism, potentially through stress-induced parafunctional habits and reduced physical activity. Dental professionals should be aware of the potential impact of gaming on oral health and consider screening for TMD symptoms in patients who are frequent gamers. Preventive strategies, including patient education on ergonomic practices, stress management, physical activity promotion, and the use of occlusal appliances, may be beneficial. Further research is needed to explore underlying mechanisms and develop targeted interventions.

## Figures and Tables

**Table 1:** Demographic characteristics and gaming habits of gamers and non-gamers.

Characteristic	Gamers (n = 48)	Non-Gamers (n = 60)	*p*-Value	Effect Size
Age (years, Mean ± SD)	21.5 ± 0.8	21.4 ± 0.9	0.6	0.10 (Cohen’s d)
Gender (%)			0.001	0.35 (Cramer’s V)
Male	62.5% (30)	10% (6)		
Female	37.5% (18)	90% (54)		
Daily Screen Time (hours, Mean ± SD)	6.5 ± 1.5	4.0 ± 1.2	<0.001	1.20 (Cohen’s d)
Daily Gaming Time (hours, Mean ± SD)	4.0 ± 1.0	N/A	N/A	N/A
Physical Activity (hours/week)	2.0 ± 1.5	3.5 ± 2.0	0.002	−0.68 (Cohen’s d)

SD—Standard Deviation; N/A—Not Applicable.

**Table 2:** Comparison of mandibular movements and pain during movements between gamers and non-gamers.

Measurement	Gamers Mean ± SD	Non-Gamers Mean ± SD	*p*-Value	Effect Size
Maximum Unassisted Opening (mm)	48.31 ± 5.91	46.33 ± 6.48	0.04	0.43 (Cohen’s d)
Pain During Unassisted Opening (%)	25% (12)	15% (9)	0.18	0.14 (phi)
Maximum Assisted Opening (mm)	53.92 ± 8.29	51.27 ± 6.87	0.05	0.38 (Cohen’s d)
Pain During Assisted Opening (%)	35% (17)	20% (12)	0.08	0.11 (phi)
Right Lateral Movement (mm)	9.19 ± 3.63	9.28 ± 6.56	0.93	0.03 (Cohen’s d)
Pain During Right Lateral Movement (%)	20% (10)	15% (9)	0.48	0.10 (phi)
Left Lateral Movement (mm)	9.39 ± 3.56	8.52 ± 3.49	0.2	0.26 (Cohen’s d)
Pain During Left Lateral Movement (%)	22% (11)	18% (11)	0.62	0.09 (phi)
Protrusion (mm)	7.82 ± 3.16	7.49 ± 8.64	0.8	0.05 (Cohen’s d)
Pain During Protrusion (%)	18% (9)	12% (7)	0.36	0.12 (phi)

SD—Standard Deviation.

**Table 3:** Comparison of palpation-induced pain in masticatory muscles between gamers and non-gamers.

Muscle/Area	Gamers Pain Present (%)	Non-Gamers Pain Present (%)	*p*-Value	Effect Size
Masseter Muscle	45.8% (22)	30.0% (18)	0.05	0.19 (phi)
Temporalis Muscle	35.4% (17)	25.0% (15)	0.2	0.15 (phi)
TMJ Lateral Pole	29.2% (14)	20.0% (12)	0.25	0.14 (phi)
TMJ Periarticular Area	22.9% (11)	15.0% (9)	0.28	0.13 (phi)
Lateral Pterygoid Region	20.8% (10)	10.0% (6)	0.11	0.20 (phi)

**Table 4:** Comparison of dental wear index scores between gamers and non-gamers across multiple teeth.

Tooth	Gamers Mean ± SD	Non-Gamers Mean ± SD	Mean Difference	*p*-Value	Effect Size (Cohen’s d)
1.3	0.58 ± 0.71	0.57 ± 0.65	0.01	0.88	0.02
1.6	0.32 ± 0.47	0.19 ± 0.40	0.13	0.14	0.45
2.3	0.68 ± 0.73	0.55 ± 0.63	0.13	0.03	0.37
2.6	0.28 ± 0.62	0.26 ± 0.55	0.02	0.85	0.06
3.3	0.58 ± 0.68	0.42 ± 0.53	0.16	0.04	0.35
3.6	0.37 ± 0.57	0.23 ± 0.43	0.14	0.16	0.34
4.3	0.56 ± 0.77	0.54 ± 0.60	0.02	0.86	0.04
4.6	0.27 ± 0.54	0.32 ± 0.51	−0.05	0.64	−0.17

**Table 5:** Independent *t*-tests comparing mandibular measurements and dental wear between gamers and non-gamers.

Measurement	Mean Difference	95% CI	t-Value	*p*-Value	Effect Size (Cohen’s d)
Maximum Unassisted Opening (mm)	1.98	0.11 to 3.85	2.07	0.04	0.43
Maximum Assisted Opening (mm)	2.65	−0.02 to 5.32	1.98	0.05	0.38
Tooth 2.3 Wear Score	0.13	0.01 to 0.25	2.17	0.03	0.37
Tooth 3.3 Wear Score	0.16	0.01 to 0.31	2.07	0.04	0.35
Physical Activity (hours/week)	−1.5	−2.45 to −0.55	−3.10	0.002	−0.68

**Table 6:** Pearson correlation coefficients between hours spent gaming and measurements.

Variable	Correlation Coefficient (r)	*p*-Value
Hours Gaming vs. Max Unassisted Opening	0.25	0.01
Hours Gaming vs. Tooth 2.3 Wear Score	0.3	0.002
Hours Gaming vs. Masseter Pain	0.22	0.02
Hours Gaming vs. Physical Activity	−0.35	<0.001

**Table 7:** Logistic regression analysis predicting the presence of TMD symptoms.

Variable	Odds Ratio (OR)	95% CI	*p*-Value
Gaming Status (Gamer)	2.5	1.1 to 5.6	0.03
Daily Screen Time (hours)	1.2	1.0 to 1.4	0.05
Physical Activity (hours/week)	0.8	0.7 to 0.95	0.01
Gender (Male)	1.3	0.6 to 2.8	0.5

## Data Availability

Data availability is subject to hospital approval.
